# Blood cadmium level as a risk factor for chronic pain: NHANES database 1999–2004

**DOI:** 10.3389/fpubh.2024.1340929

**Published:** 2024-05-21

**Authors:** Panpan Mi, Haoran Dong, Shengle Chen, Xuan Gao, Xu Cao, Yong Liu, Huijie Wang, Guofeng Fan

**Affiliations:** ^1^Department of Orthopaedic, Hebei PetroChina Central Hospital, Langfang, China; ^2^Hospital of Stomatology, Hebei Medical University, Shijiazhuang, China; ^3^Department of Endoscopy, Shijiazhuang Traditional Chinese Medicine Hospital, Shijiazhuang, China

**Keywords:** blood cadmium, chronic pain, risk factor, NHANES, cross-sectional study

## Abstract

**Objective:**

The escalating prevalence of chronic pain poses a substantial socio-economic burden. Chronic pain primarily stems from musculoskeletal and nervous system impairments. Given cadmium's known toxicity to these systems, our study sought to investigate the correlation between blood cadmium levels and chronic pain.

**Methods:**

The cross-sectional study was conducted from the National Health and Nutrition Examination Survey (NHANES, 1999–2004), and comprised US adults who participated in a chronic pain interview. We employed logistic regression models and smooth curve fitting to elucidate the relationship between blood cadmium levels and chronic pain.

**Results:**

Our findings revealed a linear association between blood cadmium levels and chronic pain. Compared to the lower blood cadmium tertile 1 (<0.3 ug/dL), the adjusted odds ratios (ORs) for tertile 2 (0.3–0.4 ug/dL), and tertile 3 (≥0.5 ug/dL), were 1.11 (0.96–1.29) and 1.2 (1.03–1.39), respectively. Sensitivity analyses corroborated these results.

**Conclusion:**

Elevated levels of blood cadmium are associated with a heightened risk of chronic pain among adults in the United States. Mitigating cadmium exposure could potentially decrease the risk of chronic pain, thereby enhancing strategies for chronic pain prevention and management.

## 1 Introduction

Chronic pain is a pervasive, complex, and often debilitating condition that is increasing in prevalence worldwide, resulting in significant economic and social consequences (1). While it is frequently a sequela of injury or disease, it is also recognized as a distinct condition in its own right, not simply a symptom of other diseases (2). Chronic pain is characterized as a persistent discomfort that surpasses the typical duration of tissue healing. According to the International Association for the Study of Pain, this period is generally considered to exceed 3 months, particularly in the absence of other contributing factors (3). Numerous risk factors are implicated in the onset of chronic pain, encompassing a broad spectrum of domains such as sociodemographic characteristics, psychological conditions, clinical manifestations, and biological attributes (4–12). A thorough understanding of these risk factors can help guide specific prevention and management strategies.

Cadmium is a well-known hazardous pollutant that is widely distributed in the environment (13). Exposure to cadmium in the environment poses a range of public health issues. In the general population, cadmium is commonly absorbed through inhalation of cigarette smoke particles or ingestion of contaminated food, such as shellfish, offal, and vegetables (14). According to previous research, cadmium can be harmful to both the musculoskeletal and neurological systems (15–18), potentially leading to chronic pain (19). Cadmium poisoning is known to cause femoral and lumbar pain initially, followed by the spread of pain to other parts of the body (14). Itai-itai disease represents an extreme manifestation of chronic cadmium toxicity (20) with back pain as a distinguishing symptom (21).

As far as we understand, no existing studies have delved into the association between chronic pain and exposure to cadmium. Consequently, we aim to utilize data from the National Health and Nutrition Examination Survey (NHANES) to conduct an in-depth investigation into the relationship between blood cadmium levels and the incidence of chronic pain among adults in the United States. Our research hypothesis posits that individuals with higher blood cadmium levels are more susceptible to chronic pain.

## 2 Materials and methods

### 2.1 Study population

The study population for this research was derived from the National Health and Nutrition Examination Survey (NHANES), a comprehensive study conducted by the National Center for Health Statistics (NCHS) of the Centers for Disease Control and Prevention (CDC). The NHANES database includes in-person interviews, laboratory tests, and clinical evaluations. Data from three consecutive NHANES survey cycles (1999–2000, 2001–2002, 2003–2004) that included both chronic pain assessment and blood cadmium level data were selected for this study.

### 2.2 Chronic pain assessment

The assessment of chronic pain was conducted for all participants aged 20 years and above through a series of interview questions. Participants who reported experiencing pain persisting for 3 months or more were classified under the chronic pain category, while the remainder were categorized as non-chronic pain sufferers.

### 2.3 Measurement of blood cadmium levels

For the years 1999–2002, an atomic absorption spectrometer was utilized for the quantification of blood cadmium levels. In contrast, for the years 2003–2004, inductively coupled plasma mass spectrometry was employed.

### 2.4 Other variables

The study analyzed several demographic factors, including age, sex, race, marital status, body mass index (BMI), poverty income ratio (PIR), and education level. Alcohol consumption was assessed by asking participants about their frequency of drinking over the previous 12 months. Smoking status was determined based on whether an individual had consumed a minimum of 100 cigarettes throughout their lifetime (22). Physical activity was divided into four categories: mainly sitting, walking around, light physical activity, and heavy physical activity. Data on cotinine levels, blood lead levels, arthritis, cancer or malignancy, coronary heart disease, and osteoporosis were available and can be accessed at http://www.cdc.gov/nchs/nhanes/. Determining if someone has diabetes involves three criteria: (1) the person reports having the condition or is taking medication for it; (2) their blood sugar level after fasting is at least 7.0 mmol/L; and (3) their HbA1c level is at least 6.5% (23). To determine if someone has hypertension, two criteria are used: (1) the person reports having the condition or is taking medication for it, and (2) the average of three systolic blood pressure readings is at least 140 mmHg or the average of three diastolic blood pressure readings is at least 90 mmHg (24).

### 2.5 Statistical analysis

Continuous data were presented either as mean ± standard deviation or median (Q1–Q3) values. A *P*-value of <0.05 was considered statistically significant (two-tailed). All data analyses were performed using the statistical software packages R 3.3.2 and Free Statistics software version 1.7.1 (25).

The relationship between blood cadmium and chronic pain was investigated using logistic regression models. Blood cadmium levels (ug/dL) were divided into three tertiles (<0.3, 0.3–0.4, and ≥0.5) to evaluate their impact. Three models were constructed: an unadjusted model, a model adjusted for demographic factors, and a model adjusted for demographic factors + alcohol consumption, body mass index, physical activity, cotinine, blood lead, and comorbidities. These potential confounders were determined based on previous studies or the results of univariate logistic regression analyses. Subgroup analyses and smooth curve fitting were performed in this study. Sensitivity analysis was conducted using complete cases. Multiple imputations (5 replications) were used to address missing data.

## 3 Results

### 3.1 Baseline characteristics

**Figure 1:**
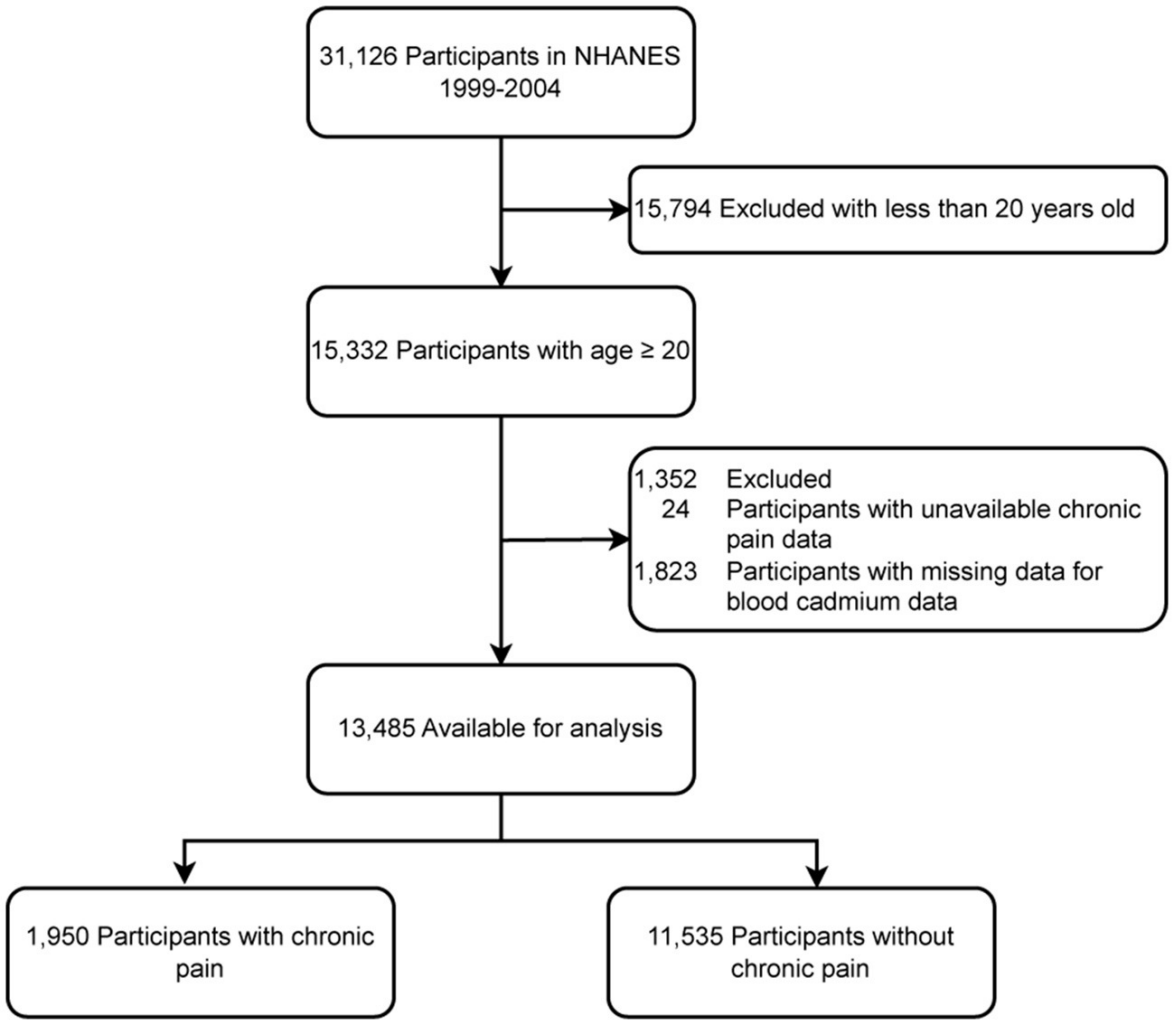
The study flowchart.

In this study, 31,126 potential participants were initially identified, but only 13,485 adults aged 20 years and above were included. Figure 1 presents the exclusion criteria for the study. Table 1 displays the characteristics of participants, categorized by their blood cadmium level. The findings suggest a higher propensity for chronic pain among individuals with elevated blood cadmium concentrations.

**Table 1:** Participants characteristics.

**Variables**	**Blood cadmium**				***P*-value**
	**Total** ***n** =* **13,485**	**Q1** ***n** =* **3,174**	**Q2** ***n** =* **4,197**	**Q3** ***n** =* **6,114**	
Chronic pain, *n* (%)	1,950 (14.5)	369 (11.6)	554 (13.2)	1,027 (16.8)	<0.001
Duration, *n* (%)					0.154
3–12 months	446 (22.9)	92 (24.9)	137 (24.7)	217 (21.1)	
>12 months	1,504 (77.1)	277 (75.1)	417 (75.3)	810 (78.9)	
Age, year	49.8 ± 19.1	41.6 ± 16.9	49.6 ± 19.0	54.2 ± 18.7	<0.001
Sex, male, *n* (%)	6,401 (47.5)	1,692 (53.3)	1,875 (44.7)	2,834 (46.4)	<0.001
BMI, kg/m^2^	28.4 ± 6.2	28.9 ± 6.7	28.7 ± 6.2	27.9 ± 6.0	<0.001
Race, *n* (%)					<0.001
Mexican American	3,060 (22.7)	682 (21.5)	1,092 (26)	1,286 (21)	
Other Hispanic	613 (4.5)	145 (4.6)	229 (5.5)	239 (3.9)	
Non-Hispanic white	6,808 (50.5)	1,653 (52.1)	2,039 (48.6)	3,116 (51)	
Non-Hispanic black	2,524 (18.7)	628 (19.8)	710 (16.9)	1,186 (19.4)	
Other races	480 (3.6)	66 (2.1)	127 (3)	287 (4.7)	
Marital status, *n* (%)					<0.001
Married or living with a partner	8,380 (62.1)	2,106 (66.4)	2,765 (65.9)	3,509 (57.4)	
Living alone	5,105 (37.9)	1,068 (33.6)	1,432 (34.1)	2,605 (42.6)	
Education level, *n* (%)					<0.001
<9 years	4,391 (32.6)	736 (23.2)	1,285 (30.6)	2,370 (38.8)	
9–12 years	3,195 (23.7)	707 (22.3)	(22.6)	1,539 (0.2)	
>12 years	5,899 (43.7)	1731 (54.5)	1,963 (46.8)	2,205 (36.1)	
PIR	2.3 (1.2, 4.2)	2.7 (1.4, 4.8)	2.5 (1.3, 4.4)	1.9 (1.1, 3.6)	<0.001
Smoking status, ≥100 cigarettes in life, *n* (%)	6,550 (48.6)	776 (24.4)	1,507 (35.9)	4,267 (69.8)	<0.001
Alcohol consumption, drink	1.0 (0.0, 3.0)	2.0 (1.0, 3.0)	1.0 (0.0, 2.0)	1.0 (0.0, 3.0)	<0.001
Physical activities, *n* (%)					<0.001
Mainly sit	3,460 (25.7)	735 (23.2)	1,035 (24.7)	1,690 (27.6)	
Walk around	7,097 (52.6)	1,614 (50.9)	2,300 (54.8)	3,183 (52.1)	
Light load	2,045 (15.2)	583 (18.4)	630 (15)	832 (13.6)	
Heavy load	883 (6.5)	242 (7.6)	232 (5.5)	409 (6.7)	
Blood lead, ug/dL	1.7 (1.1, 2.7)	1.3 (0.8, 1.9)	1.6 (1.1, 2.4)	2.2 (1.4, 3.3)	<0.001
Cotinine, ng/mL	0.1 (0.0, 12.9)	0.0 (0.0, 0.2)	0.0 (0.0, 0.2)	1.2 (0.0, 188.0)	<0.001
Blood cadmium, ug/dL	0.4 (0.3, 0.7)	0.2 (0.2, 0.2)	0.4 (0.3, 0.4)	0.7 (0.5, 1.0)	<0.001
Arthritis, *n* (%)	3,458 (25.6)	553 (17.4)	1,039 (24.8)	1,866 (30.5)	<0.001
Coronary heart disease, *n* (%)	603 (4.5)	85 (2.7)	179 (4.3)	339 (5.5)	<0.001
Cancer or malignancy, *n* (%)	1,142 (8.5)	157 (4.9)	336 (8)	649 (10.6)	<0.001
Osteoporosis, *n* (%)	742 (5.5)	91 (2.9)	221 (5.3)	430 (7)	<0.001
Hypertension, *n* (%)	5,296 (39.3)	952 (30)	1,598 (38.1)	2,746 (44.9)	<0.001
Diabetes, *n* (%)	1,773 (13.1)	380 (12)	554 (13.2)	839 (13.7)	0.06

Data are shown as mean ± SD, median (IQR), or *n* (%). Q, quartile; BMI, body mass index; PIR, poverty income ratio.

### 3.2 Relationship between blood cadmium and chronic pain

Univariate analysis (Supplementary Table 1) revealed that several factors, including age, sex, BMI, education level, race, alcohol consumption, smoking status, physical activity, and comorbidities, such as arthritis, diabetes, coronary heart disease, cancer or malignancy, hypertension, and osteoporosis, were related to chronic pain.

To evaluate the association between blood cadmium and chronic pain, multivariable logistic regression analyses were performed. The results showed that blood cadmium was positively related to chronic pain, even after adjusting for potential covariates (Table 2, Model 1, OR: 1.28 [1.18–1.38], *P* < 0.001; Model 2, OR: 1.1 [1.03–1.23], *P* = 0.01).

**Table 2:** Association of blood cadmium with chronic pain.

**Variable**	**Event, *n* (%)**	**Unadjusted model**		**Model 1** ^**a**^		**Model 2** ^**b**^	
		**OR (95% CI)**	***P*** **-value**	**OR (95% CI)**	***P*** **-value**	**OR (95% CI)**	***P*** **-value**
Blood cadmium, ug/dL	1,950/13,485 (14.5)	1.36 (1.27~1.46)	<0.001	1.28 (1.18~1.38)	<0.001	1.13 (1.03~1.23)	0.01
**Blood cadmium, tertile, ug/dL**							
<0.3	369/3,174 (11.6)	1 (Ref)		1 (Ref)		1 (Ref)	
0.3–0.4	554/4,197 (13.2)	1.16 (1~1.33)	0.043	1.09 (0.94~1.26)	0.24	1.11 (0.96~1.29)	0.172
≥0.5	1,027/6,114 (16.8)	1.53 (1.35~1.74)	<0.001	1.33 (1.16~1.52)	<0.001	1.2 (1.03~1.39)	0.018
*P* for trend			<0.001		<0.001		0.018

OR, odds ratio; CI, confidence interval. ^a^Adjusted for age, sex, marital status, poverty income ratio, race, and education level. ^b^Adjusted for Model 1 + body mass index, physical activity, alcohol consumption, cotinine, blood lead, diabetes, arthritis, cancer or malignancy, hypertension, osteoporosis, and coronary heart disease.

**Figure 2:**
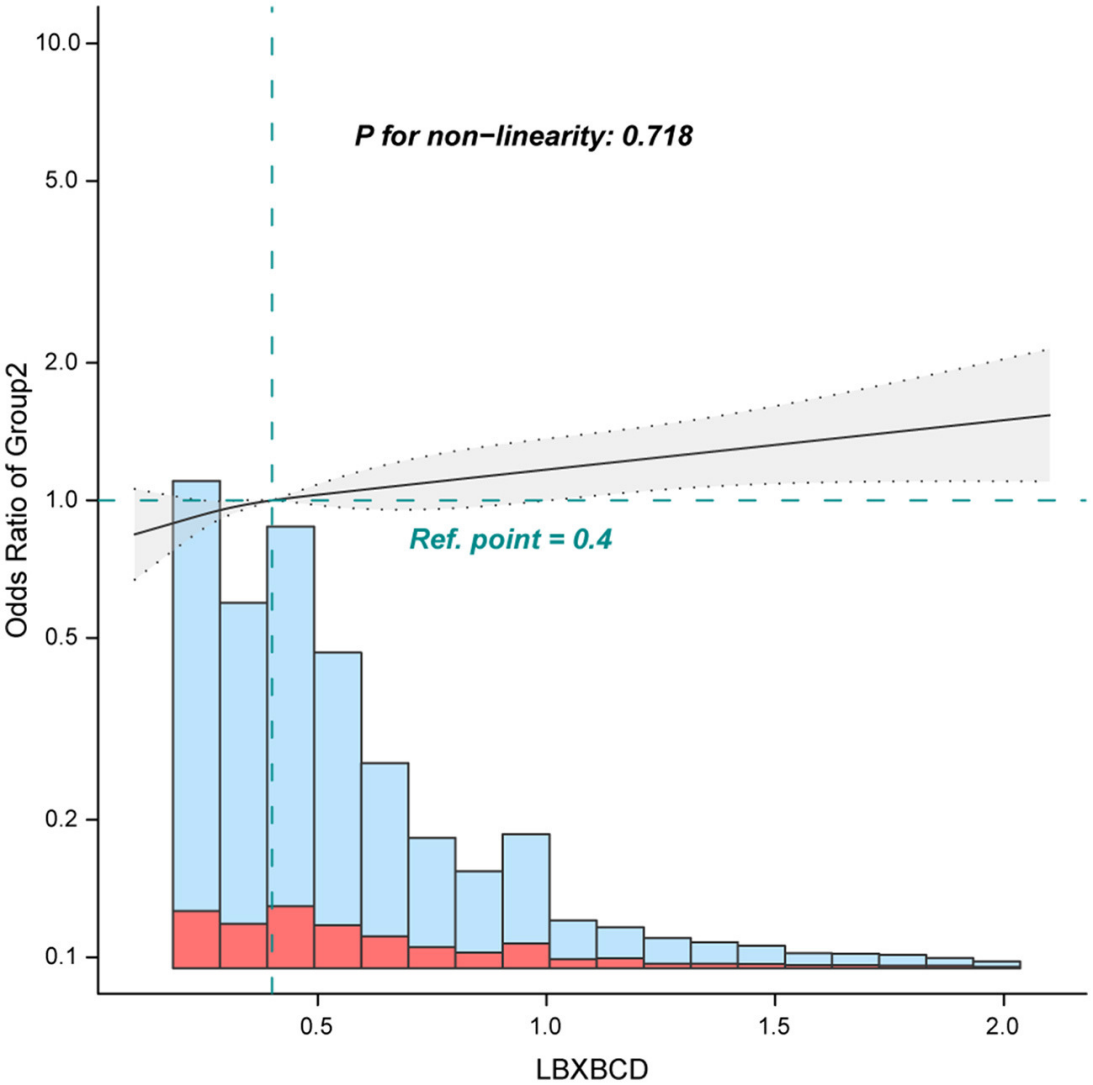
Association between blood cadmium with chronic pain. Adjusted for age, sex, marital status, poverty income ratio, race, education level, body mass index, alcohol consumption, physical activity, osteoporosis, diabetes, hypertension, cancer or malignancy, arthritis, coronary heart disease, cotinine, and blood lead. Only 97.5% of the data is shown.

When blood cadmium was analyzed in terms of tertiles, the association between blood cadmium and chronic pain was robust. The adjusted ORs for tertile 2 (0.3–0.4 ug/dL), and tertile 3 (≥0.5 ug/dL) were 1.11 (0.96–1.29) and 1.2 (1.03–1.39), respectively, compared with the lowest blood cadmium tertile 1 (<0.3 ug/dL) (Table 2, Model 2). Furthermore, the smooth curve analysis revealed a linear relationship between blood cadmium and chronic pain after adjusting for Model 2 (Figure 2, only 97.5% of the data is shown).

### 3.3 Sensitivity analysis

**Figure 3:**
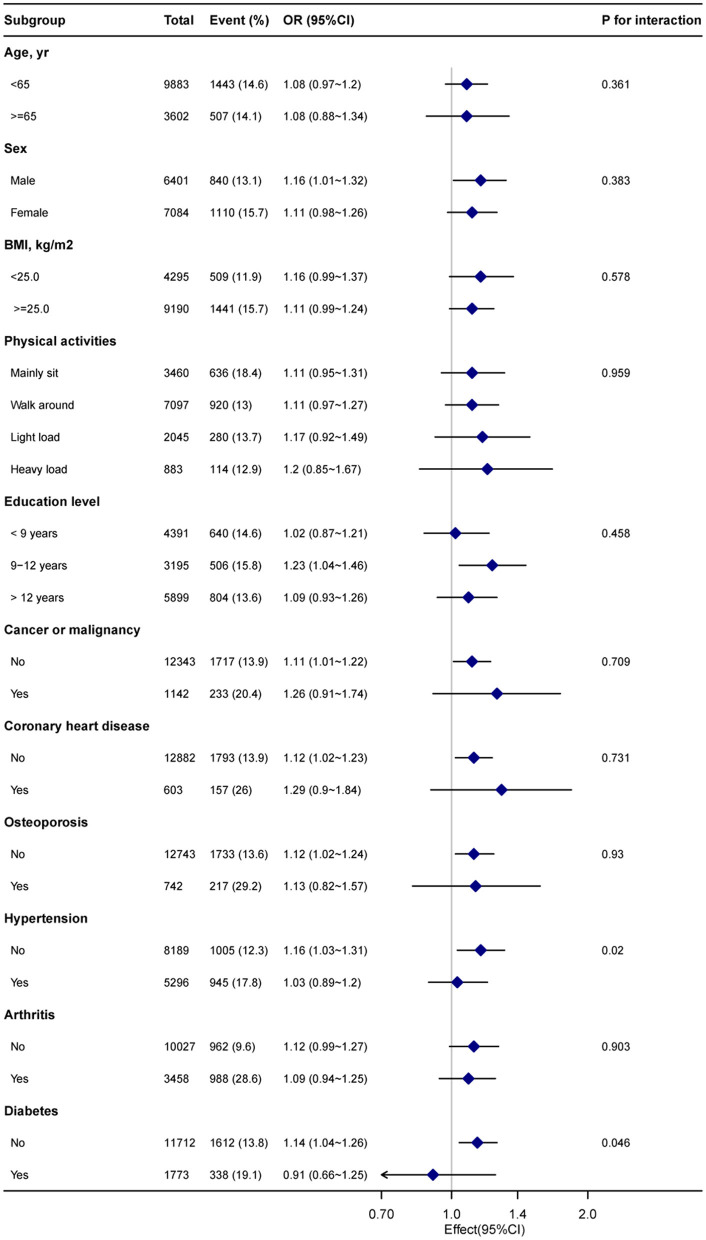
Subgroup analysis of the blood cadmium and chronic pain. Each stratification factor was adjusted for age, sex, poverty income ratio, marital status, race, education level, body mass index, alcohol consumption, physical activity, osteoporosis, diabetes, hypertension, cancer or malignancy, arthritis, coronary heart disease, cotinine, and blood lead.

Stratified analyses were performed to examine potential modifiers in the relationship between blood cadmium levels and chronic pain. Upon stratification by factors such as sex, age (<65 and ≥65 years), BMI (<25 and ≥25 kg/m^2^), physical activity, education level, and comorbidities (as depicted in Figure 3). However, considering multiple testing, the *P*-values for the interaction in hypertension (*P* = 0.02) and diabetes (*P* = 0.046) subgroups may not reach statistical significance.

Finally, the sensitivity analysis confirmed the linear association between blood cadmium and chronic pain even after excluding those with missing data (Supplementary Table 2).

## 4 Discussion

The objective of this investigation was to elucidate the relationship between blood cadmium levels and chronic pain within the adult population of the United States, utilizing the nationally representative NHANES database. Our findings revealed a positive correlation between elevated blood cadmium levels and the prevalence of chronic pain. This association remained consistent across various subgroups and sensitivity analyses, thereby underscoring the robustness of the relationship between blood cadmium concentrations and chronic pain.

Previous studies on blood cadmium did not focus on chronic pain as a disease in its own right, but rather as a comorbidity of other diseases. However, chronic pain should be considered a healthcare issue and recognized as a disease in its own right (26). Cadmium can cause various diseases accompanied by chronic pain, such as Itai-itai disease, which results from long-term exposure to cadmium-contaminated rice fields. Patients experience a gradual extension of back and hip pain to the rest of the body, which persists for several years (17). Chronic pain is also accompanied by long-term back pain, and cadmium has been significantly associated with arthritis and rheumatoid arthritis in NHANES data (27). However, large cohort studies are still required to supplement the evidence of the relationship between blood cadmium and chronic pain.

While the mechanism underlying the relationship between blood cadmium and chronic pain is not fully understood, it can be explained by cadmium's toxic effects on the skeletal and nervous systems. Cadmium exerts an inhibitory effect on bone formation and stimulates bone resorption, influencing the functional activity of osteoblasts and osteoclasts. It also substitutes calcium in hydroxyapatite crystals, which leads to disorders in calcium metabolism, bone osteoporosis, osteomalacia, and demineralization. Consequently, this results in chronic pain localized in the limbs, pelvis, and spine (28–31). Cadmium has been observed to instigate oxidative stress, augmenting H_2_O_2_ levels within bone structures, while concurrently diminishing the concentrations of glutathione peroxidase and catalase (32). Furthermore, it stimulates the generation of inflammatory mediators, known as cytokines, within the immune system, leading to significant pain (33). Moreover, cadmium serves as a potent neurotoxic agent, inflicting adverse effects on the peripheral nervous system (34), which leads to oxidative stress (35, 36) and neurogenic damage resulting in neuronal cell death (37). Nerve damage is a major cause of neuropathic pain (19).

Reducing cadmium exposure may help reduce the risk of chronic pain, but it is not easy to achieve. Occupational and environmental exposure to cadmium primarily originates from industrial activities such as battery production, metallurgy, plastic manufacturing, pigments, mining, chemical stabilizers, and metal coatings, as well as contaminated farmland and food (38). Tobacco smoke contains cadmium, which further increases human exposure to cadmium (39). Moreover, cadmium is characterized by an extended biological half-life and a sluggish elimination rate within the human organism, leading to its persistent accumulation (40). Reducing smoking and exposure to secondhand smoke are individual choices that can help reduce cadmium exposure. Identifying food contamination levels and suspected areas and considering public education and awareness programs for cadmium poisoning prevention are also recommended (14).

However, this study has several limitations. Firstly, the pain data in the NHANES database is self-reported through questionnaire surveys, which may be subject to recall bias. However, trained interviewers were used to collect the data, and these methods have been validated and consistent over many years of data collection (41). Secondly, the NHANES database only collected pain data from 1999 to 2004, and there were no more detailed pain data, such as measurements of pain intensity and functional interference. This hinders our ability to examine the intricate details of pain in correlation with blood cadmium levels. Furthermore, the NHANES dataset is collected from the U.S. population, which may lead to regional bias. Finally, retrospective studies have inherent limitations such as missing data for some variables. Nevertheless, multiple imputations were performed and the results were stable. Multivariate logistic regression and subgroup analysis were conducted to reduce residual confounding effects from unknown factors.

## Data availability statement

The raw data supporting the conclusions of this article will be made available by the authors, without undue reservation.

## Ethics statement

The studies involving humans were approved by the NCHS Research Ethics Review Board. The studies were conducted in accordance with the local legislation and institutional requirements. The participants provided their written informed consent to participate in this study.

## Author contributions

HW: Conceptualization, Methodology, Supervision, Writing – review & editing. PM: Data curation, Formal analysis, Investigation, Writing – original draft. HD: Data curation, Formal analysis, Investigation, Writing – original draft. SC: Data curation, Investigation, Writing – original draft. XG: Data curation, Investigation, Writing – original draft. XC: Data curation, Investigation, Writing – original draft. YL: Data curation, Investigation, Writing – original draft. GF: Conceptualization, Supervision, Writing – review & editing.

## References

[1] Dueñas M Ojeda B Salazar A Mico JA Failde I. A review of chronic pain impact on patients, their social environment and the health care system. J Pain Res. (2016) 9:457–67. 10.2147/JPR.S105892 27418853 PMC4935027

[2] Mills SEE Nicolson KP Smith BH. Chronic pain: a review of its epidemiology and associated factors in population-based studies. Br J Anaesth. (2019) 123:e273–e83. 10.1016/j.bja.2019.03.023 31079836 PMC6676152

[3] Raja SN Carr DB Cohen M Finnerup NB Flor H Gibson S. The revised international association for the study of pain definition of pain: concepts, challenges, and compromises. Pain. (2020) 161:1976–82. 10.1097/j.pain.0000000000001939 32694387 PMC7680716

[4] van Hecke O Torrance N Smith BH. Chronic pain epidemiology and its clinical relevance. Br J Anaesth. (2013) 111:13–8. 10.1093/bja/aet123 23794640

[5] Wiesenfeld-Hallin Z. Sex differences in pain perception. Gend Med. (2005) 2:137–45. 10.1016/S1550-8579(05)80042-7 16290886

[6] Poleshuck EL Green CR. Socioeconomic disadvantage and pain. Pain. (2008) 136:235–8. 10.1016/j.pain.2008.04.003 18440703 PMC2488390

[7] Nielsen CS Stubhaug A Price DD Vassend O Czajkowski N Harris JR. Individual differences in pain sensitivity: genetic and environmental contributions. Pain. (2008) 136:21–9. 10.1016/j.pain.2007.06.008 17692462

[8] Boersma K Linton SJ. Expectancy, fear and pain in the prediction of chronic pain and disability: a prospective analysis. Eur J Pain. (2006) 10:551–7. 10.1016/j.ejpain.2005.08.004 16199189

[9] Bär KJ Wagner G Koschke M Boettger S Boettger MK Schlösser R. Increased prefrontal activation during pain perception in major depression. Biol Psychiatry. (2007) 62:1281–7. 10.1016/j.biopsych.2007.02.011 17570347

[10] Darlow B Fullen BM Dean S Hurley DA Baxter GD Dowell A. The association between health care professional attitudes and beliefs and the attitudes and beliefs, clinical management, and outcomes of patients with low back pain: a systematic review. Eur J Pain. (2012) 16:3–17. 10.1016/j.ejpain.2011.06.006 21719329

[11] Hocking LJ Morris AD Dominiczak AF Porteous DJ Smith BH. Heritability of chronic pain in 2195 extended families. Eur J Pain. (2012) 16:1053–63. 10.1002/j.1532-2149.2011.00095.x 22337623

[12] Badraoui R Blouin S Moreau MF Gallois Y Rebai T Sahnoun Z. Effect of alpha tocopherol acetate in walker 256/b cells-induced oxidative damage in a rat model of breast cancer skeletal metastases. Chem-Biol Interact. (2009) 182:98–105. 10.1016/j.cbi.2009.09.010 19781538

[13] García-Esquinas E Téllez-Plaza M Pastor-Barriuso R Ortolá R Olmedo P Gil F. Blood cadmium and physical function limitations in older adults. Environ Pollut. (2021) 276:116748. 10.1016/j.envpol.2021.116748 33639488

[14] Rafati Rahimzadeh M Rafati Rahimzadeh M Kazemi S Moghadamnia AA. Cadmium toxicity and treatment: an update. Caspian J Intern Med. (2017) 8:135–45. 10.22088/cjim.8.3.135 28932363 PMC5596182

[15] Al-Ghafari A Elmorsy E Fikry E Alrowaili M Carter WG. The heavy metals lead and cadmium are cytotoxic to human bone osteoblasts via induction of redox stress. PLoS ONE. (2019) 14:e0225341. 10.1371/journal.pone.0225341 31756223 PMC6874340

[16] Pacini S Fiore MG Magherini S Morucci G Branca JJV Gulisano M. Could cadmium be responsible for some of the neurological signs and symptoms of myalgic encephalomyelitis/chronic fatigue syndrome. Med Hypotheses. (2012) 79:403–7. 10.1016/j.mehy.2012.06.007 22795611

[17] Reyes-Hinojosa D Lozada-Pérez CA Zamudio Cuevas Y López-Reyes A Martínez-Nava G Fernández-Torres J. Toxicity of cadmium in musculoskeletal diseases. Environ Toxicol Pharmacol. (2019) 72:103219. 10.1016/j.etap.2019.103219 31494513

[18] Wang B Du Y. Cadmium and its neurotoxic effects. Oxid Med Cell Longevity. (2013) 2013:898034. 10.1155/2013/898034 23997854 PMC3753751

[19] Cohen SP Vase L Hooten WM. Chronic pain: an update on burden, best practices, and new advances. Lancet. (2021) 397:2082–97. 10.1016/S0140-6736(21)00393-7 34062143

[20] Umemura T Wako Y. Pathogenesis of osteomalacia in itai-itai disease. J Toxicol Pathol. (2006) 19:69–74. 10.1293/tox.19.69

[21] Inaba T Kobayashi E Suwazono Y Uetani M Oishi M Nakagawa H. Estimation of cumulative cadmium intake causing itai–itai disease. Toxicol Lett. (2005) 159:192–201. 10.1016/j.toxlet.2005.05.011 16006079

[22] Hollingshead NA Vrany EA Stewart JC Hirsh AT. Differences in mexican americans' prevalence of chronic pain and co-occurring analgesic medication and substance use relative to non-hispanic white and black americans: results from nhanes 1999-2004. Pain Med. (2016) 17:1001–9. 10.1093/pm/pnv003 26814239

[23] Association AD. 2. Classification and diagnosis of diabetes: standards of medical care in diabetes-2020. Diab Care. (2020) 43:S14–S31. 10.2337/dc20-S002 31862745

[24] Zheng Y Wang J Wang Y Xu K Chen X. The hidden dangers of plant-based diets affecting bone health: a cross-sectional study with u.S. National health and nutrition examination survey (nhanes) data from 2005–2018. Nutrients. (2023) 15:1794. 10.3390/nu15071794 37049634 PMC10097387

[25] Ruan Z Lu T Chen Y Yuan M Yu H Liu R. Association between psoriasis and nonalcoholic fatty liver disease among outpatient us adults. JAMA Dermatol. (2022) 158:745–53. 10.1001/jamadermatol.2022.1609 35612851 PMC9134040

[26] Raffaeli W Arnaudo E. Pain as a disease: an overview. J Pain Res. (2017) 10:2003–8. 10.2147/JPR.S138864 28860855 PMC5573040

[27] Guan T Wu Z Xu C Su G. The association of trace elements with arthritis in us adults: Nhanes 2013-2016. J Trace Elem Med Biol. (2023) 76:127122. 10.1016/j.jtemb.2022.127122 36525916

[28] Brzóska MM Moniuszko-Jakoniuk J. Low-level lifetime exposure to cadmium decreases skeletal mineralization and enhances bone loss in aged rats. Bone. (2004) 35:1180–91. 10.1016/j.bone.2004.07.010 15542044

[29] Nambunmee K Honda R Nishijo M Swaddiwudhipong W Nakagawa H Ruangyuttikarn W. Bone resorption acceleration and calcium reabsorption impairment in a thai population with high cadmium exposure. Toxicol Mech Methods. (2010) 20:7–13. 10.3109/15376510903452941 20001568

[30] Chen X Wang G Li X Gan C Zhu G Jin T. Environmental level of cadmium exposure stimulates osteoclasts formation in male rats. Food Chem Toxicol. (2013) 60:530–5. 10.1016/j.fct.2013.08.017 23954550

[31] Charkiewicz AE Omeljaniuk WJ Nowak K Garley M Nikliński J. Cadmium toxicity and health effects—a brief summary. Molecules. (2023) 28:6620. 10.3390/molecules28186620 37764397 PMC10537762

[32] Brzóska MM Rogalska J Kupraszewicz E. The involvement of oxidative stress in the mechanisms of damaging cadmium action in bone tissue: a study in a rat model of moderate and relatively high human exposure. Toxicol Appl Pharmacol. (2011) 250:327–35. 10.1016/j.taap.2010.11.012 21129391

[33] Mahdi AA Fatima G. A quest for better understanding of biochemical changes in fibromyalgia syndrome. Indian J Clin Biochem. (2014) 29:1–2. 10.1007/s12291-013-0395-z 24478541 PMC3903931

[34] Viaene MK Roels HA Leenders J De Groof M Swerts LJ Lison D. Cadmium: a possible etiological factor in peripheral polyneuropathy. Neurotoxicology. (1999) 20:7–16. 10091854

[35] Chen L Liu L Huang S. Cadmium activates the mitogen-activated protein kinase (mapk) pathway via induction of reactive oxygen species and inhibition of protein phosphatases 2a and 5. Free Radic Biol Med. (2008) 45:1035–44. 10.1016/j.freeradbiomed.2008.07.011 18703135

[36] Chen L Xu B Liu L Luo Y Zhou H Chen W. Cadmium induction of reactive oxygen species activates the mtor pathway, leading to neuronal cell death. Free Radicals Biol Med. (2011) 50:624–32. 10.1016/j.freeradbiomed.2010.12.032 21195169 PMC3032035

[37] Son J Lee S-E Park B-S Jung J Park HS Bang JY. Biomarker discovery and proteomic evaluation of cadmium toxicity on a collembolan species, paronychiurus kimi (lee). Proteomics. (2011) 11:2294–307. 10.1002/pmic.200900690 21548089

[38] Anetor JI. Rising environmental cadmium levels in developing countries: threat to genome stability and health. Niger J Physiol Sci. (2012) 27:103–15. 10.4172/2161-0525.1000140 23652223

[39] Li H Wallin M Barregard L Sallsten G Lundh T Ohlsson C. Smoking-induced risk of osteoporosis is partly mediated by cadmium from tobacco smoke: the mros sweden study. J Bone Miner Res. (2020) 35:1424–9. 10.1002/jbmr.4014 32191351

[40] Satarug S Garrett SH Sens MA Sens DA. Cadmium, environmental exposure, and health outcomes. Environ Health Perspect. (2010) 118:182–90. 10.1289/ehp.0901234 20123617 PMC2831915

[41] Tarleton EK Kennedy AG Rose GL Littenberg B. Relationship between magnesium intake and chronic pain in US. Adults Nutr. (2020) 12:2104. 10.3390/nu12072104 32708577 PMC7400867

