# Supplementary material for: Blood cadmium level as a risk factor for chronic pain: NHANES database 1999–2004
Source: Front Public Health. 2024 May 21;12:1340929. doi: 10.3389/fpubh.2024.1340929 (PMC11148299; doi:10.3389/fpubh.2024.1340929)
Supplement: Supplementary file 1 [file Table_1.DOCX]

Table S1. Univariate analysis of risk factor associated with chronic pain.

| Variable | OR 95% CI | *P*-value |
| --- | --- | --- |
| Age, year | 1.01 (1.01~1.01) | <0.001 |
| Sex |  |  |
| Male | Ref. |  |
| Female | 1.23 (1.12~1.36) | <0.001 |
| BMI, kg/m^2^ | 1.04 (1.03~1.04) | <0.001 |
| Race |  |  |
| Mexican American | Ref. |  |
| Other Hispanic | 1.21 (0.93~1.59) | 0.16 |
| Non-Hispanic white | 1.78 (1.56~2.04) | <0.001 |
| Non-Hispanic black | 1.43 (1.21~1.68) | <0.001 |
| Other races | 1.53 (1.16~2.03) | 0.003 |
| Marital status |  |  |
| Married or living with a partner | Ref. |  |
| Living alone | 0.99 (0.9~1.1) | 0.911 |
| Education level |  |  |
| < 9 years | Ref. |  |
| 9‒12 years | 1.1 (0.97~1.25) | 0.13 |
| > 12 years | 0.92 (0.83~1.03) | 0.172 |
| PIR | 0.91 (0.88~0.94) | <0.001 |
| Smoking status |  |  |
| <100 cigarettes in life | Ref. |  |
| ≥100 cigarettes in life | 0.6 (0.54~0.66) | <0.001 |
| Alcohol consumption, drink | 0.95 (0.93~0.97) | <0.001 |
| Physical activities |  |  |
| Mainly sit | Ref. |  |
| Walk around | 0.66 (0.59~0.74) | <0.001 |
| Light load | 0.7 (0.6~0.82) | <0.001 |
| Heavy load | 0.66 (0.53~0.82) | <0.001 |
| Blood cadmium, ug/dL | 1.36 (1.27~1.46) | <0.001 |
| Blood lead, ug/dL | 1.02 (1~1.04) | 0.029 |
| Cotinine, per 10 ng/mL | 1.02 (1.01~1.02) | <0.001 |
| Diabetes |  |  |
| No | Ref. |  |
| Yes | 1.48 (1.3~1.68) | <0.001 |
| Cancer or malignancy |  |  |
| No | Ref. |  |
| Yes | 1.59 (1.36~1.85) | <0.001 |
| Coronary heart disease |  |  |
| No | Ref. |  |
| Yes | 2.18 (1.8~2.63) | <0.001 |
| Hypertension |  |  |
| No | Ref. |  |
| Yes | 1.55 (1.41~1.71) | <0.001 |
| Osteoporosis |  |  |
| No | Ref. |  |
| Yes | 2.63 (2.22~3.1) | <0.001 |
| Arthritis |  |  |
| No | Ref. |  |
| Yes | 3.77 (3.41~4.16) | <0.001 |

OR, odds ratio; CI, confidence interval; BMI, body mass index; PIR, poverty income ratio; Ref, reference.
