# Supplementary material for: Blood cadmium level as a risk factor for chronic pain: NHANES database 1999–2004
Source: Front Public Health. 2024 May 21;12:1340929. doi: 10.3389/fpubh.2024.1340929 (PMC11148299; doi:10.3389/fpubh.2024.1340929)
Supplement: Supplementary file 2 [file Table_2.docx]

Table S2. Association of blood cadmium with chronic pain.

| Variable | Event, n (%) | Crude Model | |  | Model 1^a^ | |  | Model 2^b^ | |
| --- | --- | --- | --- | --- | --- | --- | --- | --- | --- |
|  |  | OR (95% CI) | *P*-value |  | OR (95% CI) | *P*-value |  | OR (95% CI) | *P*-value |
| Blood cadmium, ug/dL | 1331/8933 (14.9) | 1.36 (1.25~1.48) | <0.001 |  | 1.26 (1.16~1.38) | <0.001 |  | 1.12 (1.01~1.25) | 0.039 |
| Blood cadmium, tertile, ug/dL | |  |  |  |  |  |  |  |  |
| <0.3 | 255/2090 (12.2) | 1(Reference) |  |  | 1(Reference) |  |  | 1(Reference) |  |
| 0.3-0.5 | 523/3784 (13.8) | 1.15 (0.98~1.35) | 0.08 |  | 1.07 (0.91~1.26) | 0.415 |  | 1.09 (0.92~1.29) | 0.333 |
| ≥0.6 | 553/3059 (18.1) | 1.59 (1.35~1.86) | <0.001 |  | 1.34 (1.13~1.59) | 0.001 |  | 1.18 (0.97~1.44) | 0.092 |
| *P* for trend |  |  | <0.001 |  |  | <0.001 |  |  | 0.09 |

OR, odds ratio; CI, confidence interval. ^a^Adjusted for sociodemographic variables (age, sex, marital status, poverty income ratio, race, and education level). ^b^Ajusted for Model 1 + body mass index, physical activity, alcohol consumption, cotinine, blood lead, diabetes, arthritis, cancer or malignancy, hypertension, osteoporosis, and coronary heart disease.
